# Functional Outcomes After Single-Bundle Anterior Cruciate Ligament Reconstruction: A Comparative Analysis of Hamstring and Peroneus Longus Autografts

**DOI:** 10.7759/cureus.95665

**Published:** 2025-10-29

**Authors:** Rajat Kapoor, Brijesh Sharma, K S Dinkar, Yashvardhan Sharma, Vivek Kumar Gautam, Amit Agarwal, Vikas K Singh

**Affiliations:** 1 Orthopedics, Sarojini Naidu (SN) Medical College, Agra, IND; 2 Orthopedics and Trauma, Sarojini Naidu (SN) Medical College, Agra, IND

**Keywords:** acl, aclr, arthroscopy, hamstring graft, peroneus longus graft

## Abstract

Background: The anterior cruciate ligament (ACL) is frequently injured in activities that involve pivoting the knee. With the development of arthroscopic techniques for reconstructing the ACL, researchers have been exploring improved graft options. This study compares the functional outcomes of two different graft sources: peroneus longus (PL) graft and hamstring graft.

Methodology: This prospective cohort study included 56 single-bundle arthroscopic ACL reconstruction patients, randomized into the PL or hamstring graft groups. Standardized surgical techniques were used, and both groups followed the same rehabilitation protocol after surgery. Knee functional outcomes were measured using International Knee Documentation Committee (IKDC) and Lysholm scores, and clinical evaluations included anterior drawer, Lachman, and pivot shift tests.

Results: Results indicated no significant differences (p > 0.05) in functional outcomes measured by IKDC and Lysholm scores between the two graft types. Additionally, graft harvest time was significantly shorter, and graft diameter was significantly larger for PL grafts.

Conclusion: Both hamstring and PL grafts are effective for ACL reconstruction, enhancing knee stability and function with minimal donor site issues. The PL tendon, with its greater strength, thickness, and minimal donor site morbidity, proves to be a highly effective and safe autograft option.

## Introduction

The anterior cruciate ligament (ACL) is a crucial stabilizer of the knee joint, and its rupture is a common and debilitating injury, particularly among active individuals [1]. The surgical reconstruction of the ACL has become the standard treatment to restore knee stability and function, enabling individuals to return to their desired level of activity [2]. The selection of an appropriate graft for ACL reconstruction is a critical determinant of the procedure's success [2].

Traditionally, hamstring (HT) tendon autografts have been widely used due to their ease of harvest and comparable biomechanical properties to the native ACL [3]. However, HT autografts are associated with potential donor site morbidity, including HT weakness and anterior knee pain, which can impact functional outcomes and patient satisfaction [4]. The quest for alternative graft options with reduced donor site morbidity and comparable or superior functional outcomes has led to growing interest in the peroneus longus (PL) tendon (PLT) as a viable alternative [5].

The PLT possesses several attributes that make it an appealing choice for ACL reconstruction. It exhibits adequate size and biomechanical strength, demonstrating sufficient resilience for knee stabilization [6]. Moreover, harvesting the PLT is associated with minimal donor site morbidity, as the peroneus brevis tendon can effectively compensate for the loss of the PLT in maintaining ankle function [7,8]. Several studies have reported encouraging results with PLT autografts, demonstrating good functional outcomes and knee stability comparable to HT autografts [5,9,10].

Despite the potential advantages of PLT autografts, the literature directly comparing their functional outcomes and donor site morbidity to HT autografts remains limited. The existing studies have primarily focused on short-term follow-up and have not comprehensively evaluated various aspects of functional recovery and patient satisfaction [10-12]. Therefore, there is a pressing need for further research to establish the long-term efficacy and safety of PLT autografts in comparison to the well-established HT autografts.

The present study aims to conduct a comparative analysis of functional outcomes, knee stability, donor site morbidity, and thigh muscle wasting in patients undergoing ACL reconstruction with either HT or PL autografts. By evaluating these parameters at various time points postoperatively, we seek to provide valuable insights into the optimal graft choice for ACL reconstruction, considering both functional recovery and patient-reported outcomes. We hypothesized that patients undergoing ACL reconstruction with PL autografts would achieve comparable functional outcomes to those with HT autografts, without significant compromise in ankle function. This study was designed to test this hypothesis through a prospective comparative analysis.

## Materials and methods

This randomized prospective study was conducted over a period of two years (2022 to 2024) and included 56 subjects who fulfilled the inclusion criteria and underwent ACL reconstruction using either HT or PL grafts. Patients aged 18-60 years with a symptomatic ACL tear confirmed on MRI and a normal contralateral knee were included in the study. Patients with previous knee surgery, multi-ligamentous injuries, meniscal tears requiring repair, fractures, osteoarthritis, systemic diseases, or medical conditions that could compromise their ability to undergo surgery or participate in rehabilitation were excluded from the study. A thorough preoperative evaluation was carried out and included demographic data, mechanism of injury, duration of injury, thigh circumference, knee stability assessment (anterior drawer [13], Lachman [13], and pivot shift [13] tests), International Knee Documentation Committee (IKDC) score [14], Lysholm score [15], and ankle-hind foot score (AOFAS) [16].

**Figure 1:**
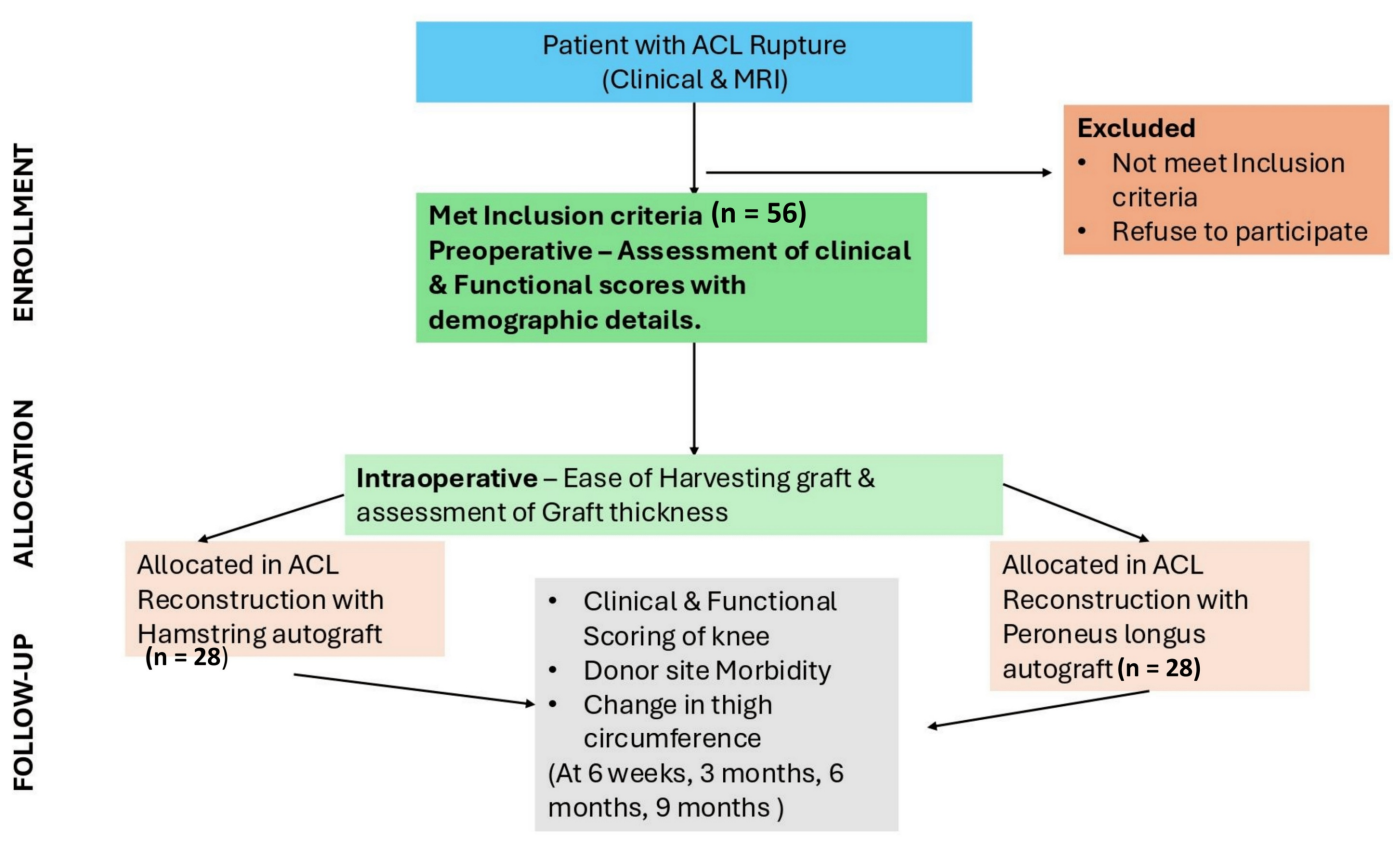
A flowchart of patient enrollment, allocation, and follow-up The CONSORT chart ACL: anterior cruciate ligament; CONSORT: Consolidated Standards of Reporting Trials

**Figure 2:**
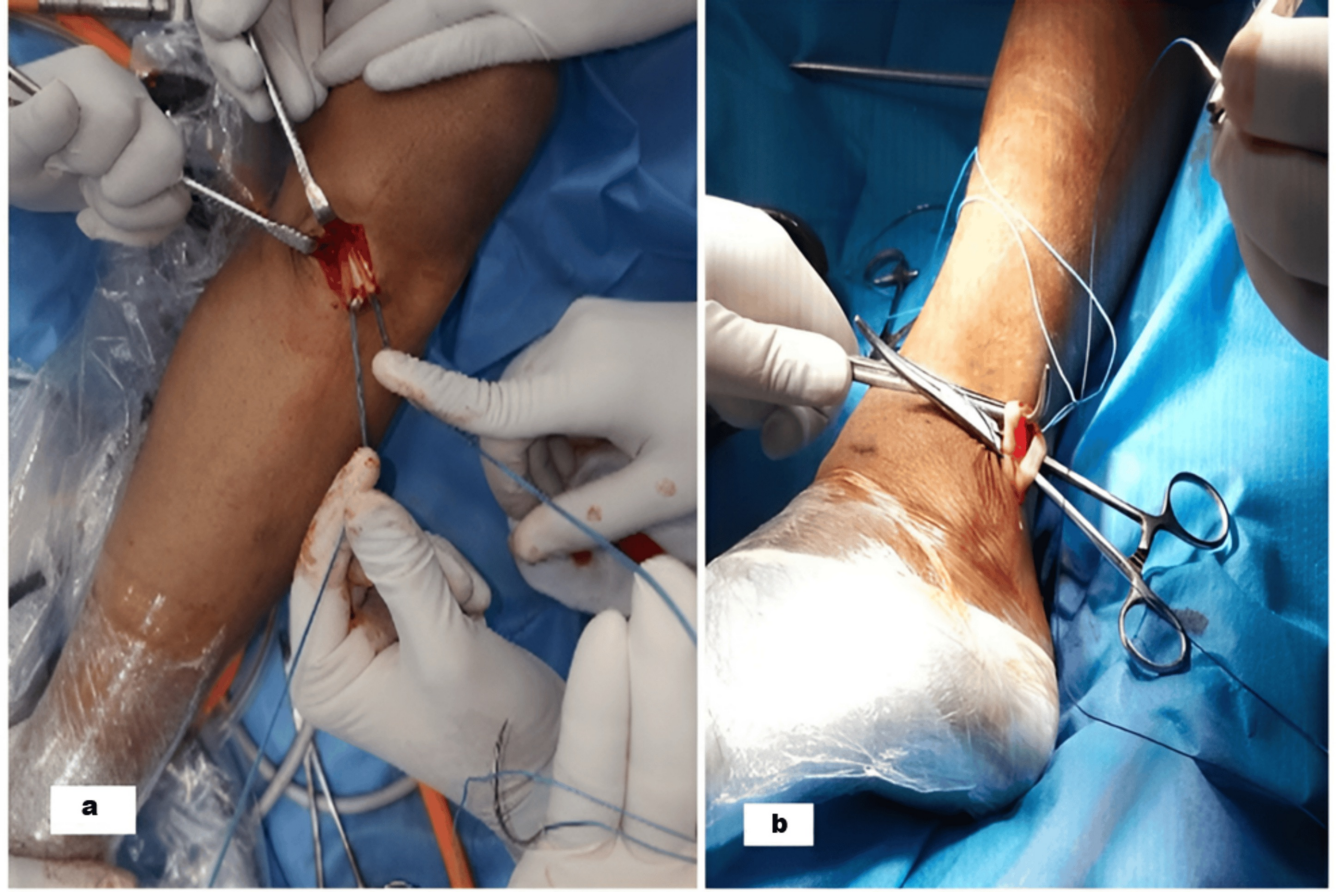
(a) Hamstring tendon graft harvesting; (b) peroneus longus tendon graft harvesting Image credit: Dr. Vivek Kumar Gautam

Figure 1 presents the CONSORT (Consolidated Standards of Reporting Trials) chart [17] for this study. The 56 patients were systematically randomized into two groups (28 (50%) each) using a computer-generated sequence, with allocation concealment strictly maintained: In group A, semitendinosus and gracilis tendons were harvested and prepared into a four-strand graft (Figure 2a). In group B, the full-thickness PLT was harvested and prepared into a two- or three-strand graft depending on the tendon's size (Figure 2b).

The procedure followed standard operative protocols. Patients were positioned in the supine position, and the surgery was performed under spinal anesthesia with a thigh tourniquet applied. Anteromedial and anterolateral portals were utilized for knee arthroscopy. Femoral fixation was achieved using an endobutton (Smith & Nephew, Andover, MA, USA), while tibial fixation was secured with a BioScrew (Smith & Nephew, Andover, MA, USA) that was one size larger than the diameter of the tibial tunnel.

A standardized rehabilitation protocol [18] (as shown in Table 1) was initiated for both groups on the first day after surgery to ensure optimal recovery. Regular follow-ups were conducted for up to nine months. Clinical evaluations included the anterior drawer [13], Lachman [13], and pivot shift [13] tests, functional outcomes were assessed using IKDC [14] and Lysholm [15] scores, knee and ankle range of motion were measured, donor site morbidity was evaluated with the AOFAS score [16], and differences in thigh girth were recorded. Assessments were made at each visit (six weeks, three months, six months, and nine months).

**Table 1:** Postoperative rehabilitation protocol ROM: range of motion

Timeline	Rehabilitation protocol
0–2 weeks	Pain and swelling control, knee flexion up to 90°, weight-bearing as tolerated with crutches and brace, quadriceps and hamstring activation.
2–6 weeks	Full ROM by 6 weeks, discontinue crutches, continue quadriceps and hamstring strengthening, prone hangs, partial squats.
6 weeks–3 months	Comfortable full ROM, brace removal, return to light pre-injury activities, slow running, wall slides, single-leg squats, static cycling.
3–6 months	Good quadriceps and hamstring strength, resistance exercises, unsupported partial squats, jogging 5–10 min.
6–8 months	Non-pivoting, non-contact sports as tolerated, continued strengthening and agility exercises.
8–10 months	Pivoting, non-contact sports as tolerated.
Beyond 10 months	Full return to all sports.

Statistical analysis was conducted using IBM SPSS Statistics software, version 25.0 (IBM Corp., Armonk, NY, USA), with Microsoft Excel 2019 (Microsoft Corp., Redmond, WA, USA) used for data organization. Preoperative data were compared with measurements taken at six weeks, three months, six months, and nine months, using paired t-tests and chi-squared tests as appropriate. The study was conducted after obtaining approval from the Institutional Ethics Committee of Sarojini Naidu (SN) Medical College, Agra (Ref. No.: SNMC/IEC/2024/259; Date: May 15, 2024). Written informed consent was obtained from all participants before enrollment in the study.

## Results

Among the participants, the majority of ACL injuries occurred in the 20-40-year age group, comprising 38 (67.86%) of cases. This was followed by the <20-year group (13 (23.21%)) and the >40-year group (5 (8.93%)). Most ACL injuries were observed in men, accounting for 51 (91.07%) of cases, while women represented five (8.93%). The primary cause of ACL injuries was road traffic accidents, which accounted for 35 (62.50%) of cases. Sports injuries were the second most common cause, comprising 18 (32.14%), while falls were relatively less frequent at three (5.36%). The mean duration of injury for the HT graft group was 16.54 weeks, compared to 17.11 weeks for the PLT graft group (Table 2).

**Table 2:** Patient demographics *p-value indicates statistical significance (p < 0.05). PL: peroneus longus

Category	Frequency (n = 56)	Hamstring graft (n = 28)	PL graft (n = 28)	Chi square/t-value	p-value
Age group	-	-	-	-	-
<20 years	13 (23.21%)	6 (21.43%)	7 (25.00%)	-	0.871
20–40 years	38 (67.86%)	19 (67.86%)	19 (67.86%)	Chi square: 0.277	-
>40 years	5 (8.93%)	3 (10.71%)	2 (7.14%)	-	-
Sex	-	-	-	-	-
Male	51 (91.07%)	25 (89.29%)	26 (92.86%)	Chi square: 4.459	0.035*
Female	5 (8.93%)	3 (10.71%)	2 (7.14%)	-	-
Mode of injury	-	-	-	-	-
Fall on the ground	3 (5.36%)	1 (3.57%)	2 (7.14%)	-	-
Road traffic accident	35 (62.50%)	20 (71.43%)	15 (53.57%)	Chi square: 1.936	0.379
Sports injury	18 (32.14%)	7 (25.00%)	11 (39.29%)	-	-
Duration of injury (weeks)	-	16.54 (SD: 5.75)	17.11 (SD: 4.18)	t-value: 0.424	0.673

The PL grafts demonstrated a significantly shorter mean graft retrieval and preparation time (7.30 minutes, SD = 0.86) compared to the HT group (12.19 minutes, SD = 1.96), with a p-value of <0.0001, reflecting a statistically significant difference (Table 3). Additionally, the PL grafts exhibited a significantly greater mean graft diameter (9.07 mm, SD = 0.57) relative to HT grafts (8.11 mm, SD = 0.21), with a p-value of <0.0001 (Table 4).

**Table 3:** Mean graft retrieval and preparation time (in minutes) *p < 0.0001 indicates a significantly shorter graft harvest time for the PL group. PL: peroneus longus

Graft used	Frequency	Mean graft retrieval and preparation time	SD	t-value	p-value
Hamstring	28	12.19	1.96	-4.890	<0.0001*
PL	28	7.30	0.86		

**Table 4:** Mean graft diameter *p < 0.0001 indicates a significantly larger graft diameter in the PL group. PL: peroneus longus

Graft used	Frequency	Mean graft diameter (in mm)	SD	t-value	p-value
Hamstring	28	8.11	0.21	8.363	<0.0001*
PL	28	9.07	0.57		

**Figure 3:**
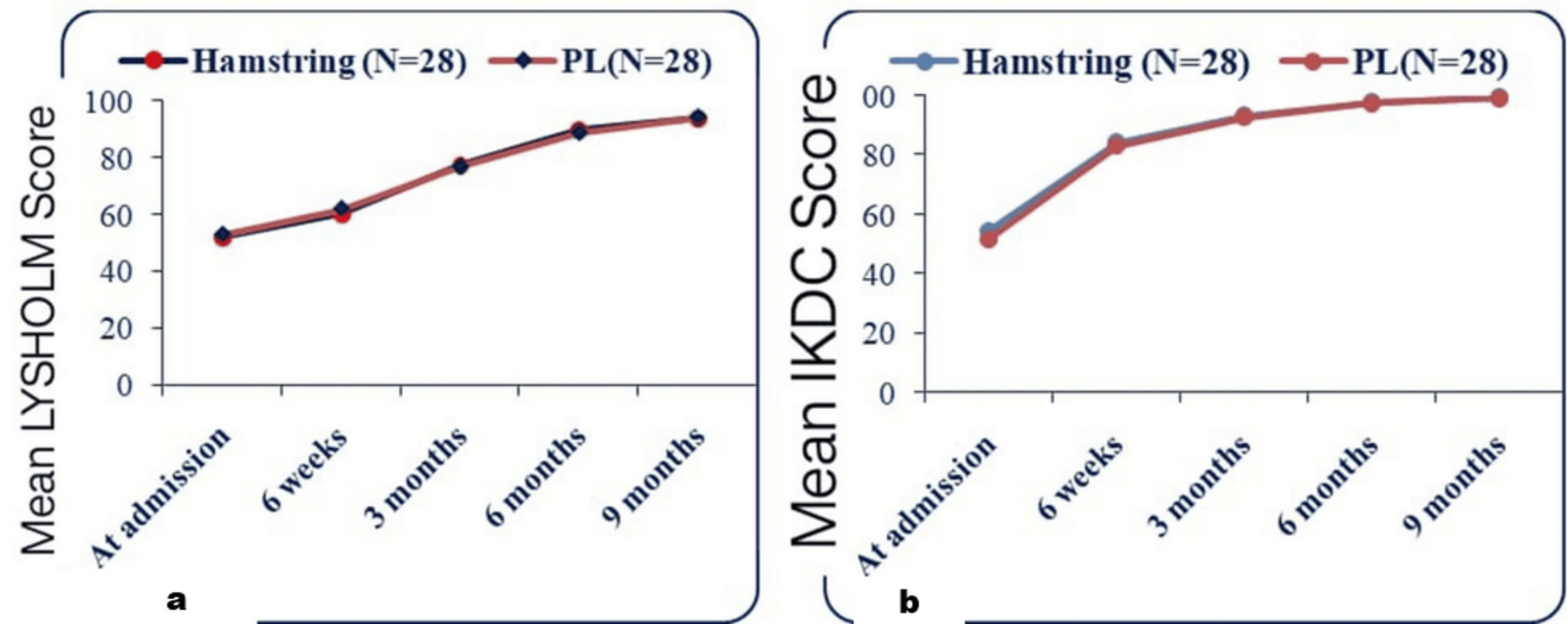
Comparison of mean Lysholm scores (a) and IKDC scores (b) between the HT and PLT graft groups HT: hamstring; PLT: peroneus longus tendon; IKDC: International Knee Documentation Committee; PL: peroneus longus

No significant differences in functional outcomes were observed between HT and PL grafts for ACL reconstruction, with comparable IKDC and Lysholm scores reported for both graft types (Figure 3).

There were no statistically significant differences in thigh circumference between the HT and PL graft groups across the assessed time periods (Table 5), suggesting comparable outcomes in terms of thigh muscle recovery following ACL reconstruction.

**Table 5:** Postoperative differences in thigh circumferences between the HT and PLT graft groups No statistically significant differences were observed between groups at any time point. HT: hamstring; PLT: peroneus longus tendon; PL: peroneus longus

Difference in thigh circumference	Hamstring (N = 28)		PL (N = 28)		t-value	p-value
	Mean	SD	Mean	SD		
At admission-6 weeks	0.72	1.99	0.65	2.01	-0.131	0.8963
At admission-3 months	2.17	2.40	1.90	2.40	-0.421	0.6755
At admission-6 months	3.40	2.73	2.88	2.75	-0.710	0.4807
At admission-9 months	4.37	3.21	3.90	3.12	-0.556	0.5808

The AOFAS scores revealed a significant initial decline for the PL graft group compared to HT grafts, but by nine months, both groups showed no significant difference in recovery outcomes (Table 6).

**Table 6:** Comparison of ankle–hind foot score (AOFAS score) between the HT graft and PLT groups *p < 0.0001 indicates a significantly lower AOFAS score in the PL group at 6 weeks, which equalized by later follow-ups. PL: peroneus longus; HT: hamstring; PLT: peroneus longus tendon

-	Hamstring (N = 28)		PL (N = 28)		t-value	p-value
	Mean	SD	Mean	SD		
At admission	100	0.00	100	0.00	-	-
6 weeks	99.86	0.76	91.57	4.06	-10.620	<0.0001*
3 months	99.45	0.85	99.25	1.27	-0.693	0.4916
6 months	99.93	0.26	99.75	0.44	-1.864	0.0678
9 months	99.93	0.26	99.99	0.32	0.770	0.4446

The study evaluated ACL reconstruction stability using the anterior drawer, Lachman, and pivot shift tests over nine months. All tests showed consistent stability (grade 0) in both HT and PL graft groups, indicating effective restoration and maintenance of knee joint stability throughout the follow-up period. Hypoesthesia near the incision site was observed in six patients (21.43%) who underwent ACL reconstruction using a HT graft. No such complication was reported in the PLT group.

## Discussion

This study shows that ACL injuries are most common in individuals aged 20-40 years (38 (67.86%) of cases), aligning with Dwidmuthe et al. [19], who reported 58.33% of participants aged 17-30 years. Other studies, such as Keyhani et al. [20] and Rhatomy et al. [5], also reported similar age ranges. These findings suggest that younger individuals, who are more physically active, are more prone to ACL injuries.

ACL injuries were predominantly seen in men (51 (91.07%)), consistent with other studies like Dwidmuthe et al. [19] and Keyhani et al. [20]. This trend highlights the higher incidence of ACL injuries among men, likely due to their higher participation in high-impact activities.

Most ACL injuries in this study were caused by road traffic accidents (35 (62.50%)), followed by sports injuries (18 (32.14%)) and falls (3 (5.36%)). This supports the study by Agarwal et al. [21], who also identified road traffic accidents as a significant cause of ACL injuries. In contrast, Rhatomy et al. [5] found that sports injuries were more common. The findings emphasize the importance of road safety and preventive measures in sports to reduce ACL injuries.

PLT grafts had a larger diameter (9.34 mm) compared to HT grafts (8.54 mm), consistent with He et al. [22] and Dwidmuthe et al. [19]. This suggests PLT grafts may offer better mechanical stability. The study also found that HT grafts required more time for retrieval and preparation than PLT grafts, supported by Agarwal et al. [21] and Dwidmuthe et al. [19]. PLT grafts may be preferable for their efficiency and safety.

Clinical tests like the anterior drawer, Lachman, and pivot shift tests showed consistent knee stability across all time points, indicating effective ACL reconstruction. This finding aligns with Vijay et al. [23], who also observed improved knee stability postoperatively.

No significant differences in functional outcomes were found between HT and PL grafts, with both showing comparable IKDC and Lysholm scores, supported by He et al. [22] and Dwidmuthe et al. [19]. This suggests PL grafts are a viable alternative to HT grafts in ACL reconstruction.

Regarding thigh circumference, no significant differences were observed between HT and PL grafts postoperatively, consistent with Dwidmuthe et al. [19] and Ambulgekar et al. [24]. Proper rehabilitation is crucial for minimizing muscle atrophy in both graft types. AOFAS scores showed excellent outcomes for both HT and PL grafts at nine months post-surgery, with no significant differences between the groups, aligning with He et al. [22] and Dwidmuthe et al. [19].

In this study, hypoesthesia was observed in 21.4% of patients with HT grafts, comparable to He et al. [22] and Dwidmuthe et al. [19]. This suggests hypoesthesia is a common complication, though its occurrence varies, emphasizing the need for careful surgical techniques to reduce nerve damage.

Limitations of the study

The small sample size of 56 may limit generalizability and statistical power, while self-reported scores like IKDC and Lysholm may introduce subjective bias, as these measures depend on patient perception and reporting accuracy. Additionally, the follow-up duration of nine months, while sufficient to assess short-term outcomes, may not capture the full long-term effects of the procedures. Due to the limited sample size, subgroup analyses by age, gender, or injury type could not be performed. Potential confounding factors, such as variability in patient activity levels, pre-existing comorbidities, and differences in rehabilitation adherence, were not controlled for and could influence functional outcomes. Further research with larger sample sizes and longer follow-up periods is warranted to confirm and expand upon these findings.

## Conclusions

The study concludes that both HT and PL grafts are effective for ACL reconstruction, enhancing knee stability and function with minimal donor site issues. The PLT, with its greater strength, thickness, and minimal donor site morbidity, proves to be a highly effective and safe autograft option. The choice between the two grafts may depend on surgeon preference and patient-specific factors. The study emphasizes the importance of proper rehabilitation for optimal recovery and highlights the need for further research with larger sample sizes and longer follow-up periods to confirm and expand upon these findings.
